# The Lived Experience of Healthcare Workers in Preventing Falls in Community Dwelling Individuals with Dementia

**DOI:** 10.3390/geriatrics7050113

**Published:** 2022-10-07

**Authors:** Nansi Felton, Toity Deave

**Affiliations:** 1 Avon and Wiltshire Mental Health Partnership NHS Trust, Bath NHS House, Newbridge Hill, Bath BA1 3QE, UK; 2 School for Health and Social Well-Being, University of the West of England, Bristol BS16 1DD, UK

**Keywords:** falls, cognition, dementia

## Abstract

Older adults living with dementia have at least twice the risk of falling compared to their peers living without cognitive impairment. There is evidence for the effectiveness of standard interventions in falls prevention in community dwellings, but they may not translate to individuals with Mild Cognitive Impairment (MCI) or dementia. A qualitative enquiry, adopting an interpretive research design underpinned by a phenomenological approach using semi-structured interviews with four healthcare workers from the field was adopted. Data were analysed using Interpretive Phenomenological Analysis to identify themes. Four major themes were developed: on-going assessment is important in guiding interventions and influencing change, knowledge and experience informs practice, individuals living with dementia have complex physical and cognitive needs, and teamwork is essential in falls prevention strategies, which highlighted falls prevention in this context being multifactorial and complex. The findings found that combining physical and cognitive strategies as part of falls prevention has potential benefits for this population, including reducing falls risks and maintaining function. Targeted training and awareness raising within a supportive multi-disciplinary team structure is required, underpinned by on-going, person-centred assessments.

## 1. Introduction

Individuals living with dementia experience progressive and irreversible loss of memory and other cognitive functions together with motor and balance impairments. Mild Cognitive Impairment (MCI) is a condition whereby someone has minor problems with cognition, including memory and thinking, but are not severe enough to interfere significantly with daily life [1]. MCI comprises of the beginnings of cognitive dysfunctions, which progress to dementia at a rate of 10% per year [2]. The neuro-psychological and physical deficits in MCI are similar to that in early dementia and the distinction between them is arbitrary [2]. Individuals living with MCI and early dementia retain many functional abilities but are at risk of deterioration, which includes the increasing risk of falls. The underlying falls risk factors related to this population are not fully understood, and may include risk factors that are unique to this condition [3,4].

In an ever-increasing older population, the number of individuals living with dementia is set to rise and dementia has been recognised as a public health priority. Falls in this population have significant social, economic, psychological, and physical consequences [5]. Additionally, people with dementia and a history of falls are five times more likely to be institutionalised [4] and are at a greater risk of sustaining injury from a fall [6]. Given the increased risks and consequences of falls in people with MCI/early dementia it is imperative that practitioners in the field can devise effective interventions based on comprehensive evidence [7]. 

Falls prevention interventions in older adults in community dwellings are well established and represented in national guidelines, but such interventions may not translate to individuals with MCI/dementia [8]. Interventions and guidelines specifically for this population are lacking [9]. Physical activity, including multicomponent exercise, for example, has been recognised to improve physical and cognitive functions and so contribute to falls prevention in the older population [10]. However, such interventions have not converted as successfully in cognitively impaired adults, and falls risk factors specific to dementia need to be considered [11]. NICE Guidelines [12], for example, identify cognitive impairment as a falls risk and also acknowledges the lack of evidence in providing effective strategies for preventing falls in this population. There is currently a global initiative project studying falls prevention in older adults, identifying the importance of including cognition in falls risk stratification and ‘how to represent this in practice guidelines’ [13] (p. 1505).

Physical exercise and cognitive training have been established as effective interventions in falls prevention as part of a multifactorial approach. However, how this translates in the context of cognitive impairment or dementia is not clear [14,15]. Additionally, combining physical and cognitive training in falls prevention lacks evidence [7], particularly in qualitative designs, which offer the opportunity to explore in-depth views and perceptions to illustrate what this would mean in clinical practice. The aim of this study is to explore and characterise the lived experience of community healthcare workers using physical and cognitive approaches as part of falls prevention strategies for individuals living with MCI or early stage dementia.

## 2. Materials and Methods

### 2.1. Study Design

This study adopted a qualitative methodology based on an interpretive phenomenological approach using semi-structured interviews.

**Figure 1:**
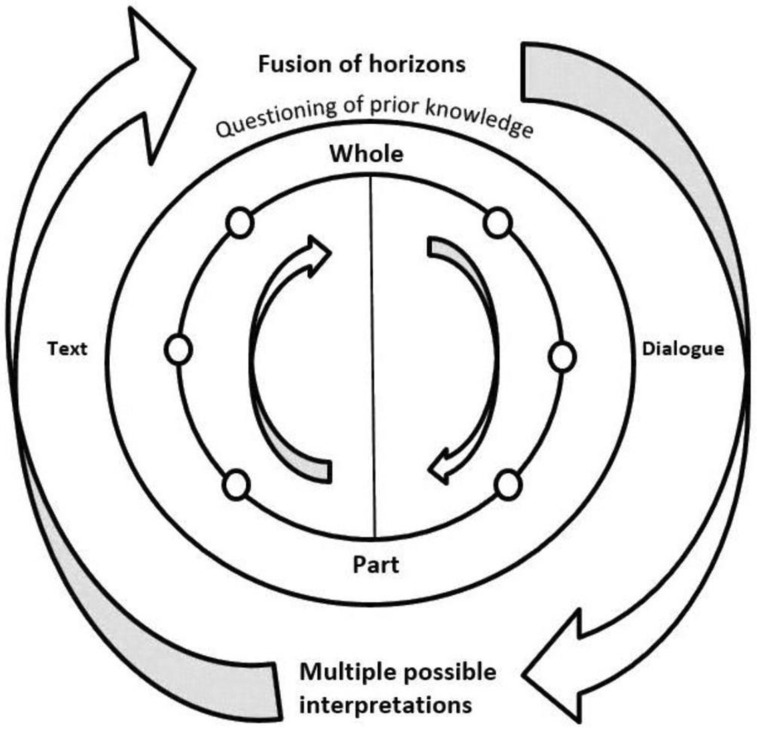
The Hermeneutic Circle, Peat et al., 2019 [16] (p. 9).

Phenomenology has gained prominence in health care as a means of exploring and interpreting complex phenomena in real-life contexts. The goal of phenomenology is not to describe but rather to explore lived experience in relation to the phenomenon being studied. Interpretive Phenomenological Analysis (IPA) can be defined as a qualitative research design with an idiographic philosophy. It focuses on the lived experiences of individuals through a process of reflective inquiry [16]. It was developed as an alternative to descriptive phenomenology by rejecting the concept of ‘bracketing’ in which the researcher puts aside any presuppositions or preconceptions they have. Using a hermeneutic approach, interpretative phenomenology recognises the implications of researcher views on this process and on the interactions with the researched [17]. The researcher is, therefore, an integral part of the research process and, rather than bracketing pre-conceptions, the researcher’s understandings are interwoven with those of the participants [18]. This is achieved through the process of the double hermeneutic circle, leading to a co-created interpretation of data (Figure 1). The process is iterative, involving moving between parts and the whole, between questions and answers, between implicit and explicit understanding, which deepens with repeated engagement [16,19].

IPA was used in this study to explore health professional’s experience of falls prevention strategies for individuals with MCI/early dementia. The approach enabled the exploration of this complex challenge, which is under-researched and provided a means whereby healthcare workers can learn from each other in co-creation of understanding and interpretation [20].

### 2.2. Participants and Recruitment

This study was conducted in South Gloucestershire in the South West of England, UK. A convenience sampling technique was used to draw from a community therapies team. The team provides specialist assessment, care. and support for older adults with complex mental health needs who live in the community, to facilitate safe and independent living for as long as possible. This team of multi-professionals works in the community caring for individuals with dementia, thus capturing the knowledge and experience necessary to respond to the research questions. The research team aimed to achieve a sample size of between 3 and 6 due to the volume intensive nature of the data and the time constraints imposed on the study.

The recruitment strategy involved a presentation delivered by NF as part of a staff Continued Professional Development (CPD) away day. The presentation outlined the study, inviting volunteers to take part, targeting relevant participants. Participant Information Sheets were distributed to accompany the presentation. Inclusion criteria were healthcare workers with experience of working with older adults with dementia in the community, including consideration of falls prevention. A total of 10 members of staff were invited to take part and 4 volunteers came forward and all were interviewed. The 4 volunteers were from different professions/roles, therefore providing rich data. There was no adverse consequence for non-participation. NF managed and completed the recruitment process that included gaining written consent from those who agreed to take part. All 4 volunteers completed all aspects of the study, with no dropouts.

### 2.3. Data Collection

The primary method of data collection was semi-structured interviews with the intension of exploring individual experience of community health care workers around falls prevention, based on the nature of their work. This format facilitated and supported the purpose of gaining a shared understanding of the phenomenon under investigation [19].

Informed consent was gained prior to any data collection. Interviews were conducted at a location and time convenient to each participant. The pre-set semi-structured interview questions were developed in relation to the primary research aims and the primary topics of interest (Table S1). The pre-set questions included broader enquiries around falls, facilitating exploration of whether or how a combined intervention approach may be incorporated as a component of a falls prevention strategy. During the interviews greater weighting was placed on exploring a combined physical and cognitive approach, therefore remaining focused on the research aims. An interview prompt guide was based on prominent topic themes from existing literature together with topics or issues considered important by the researcher from their previous research and work experience. The interview schedule was checked by a second reviewer, TD, and by clinical representations from the field to ensure relevance. Single, audio-recorded interviews were conducted with consent, each lasting up to 30 min. The researcher used field notes to document decisions and thoughts before, during and after each interview; this contributed to the data analysis and added rigour and transparency to the study [21,22]. Field notes provided a source of rich reflection and added context, which contributed to empathy, understanding, and meaning of data.

### 2.4. Data Analysis

The interviews were transcribed and analysed by NF to identify key themes or repeated words or phrases (Table S2). Transcripts were stored using Microsoft Word processing software, omitting personal data.

Phenomenological analysis is an iterative process, and is fluid and multi-directional. An inductive thematic analytical approach was used to group words or themes into clusters based on their similarities. The process was structured and guided by applying the phases of IPA (Table 1). These phases were not linear and frequently overlapped, being within the framework of the hermeneutic circle.

Analysis engagement required the researcher to become embedded in the data, closely examining the text and the language used. Analysis involved using colour-coding on each transcript to differentiate emerging themes. Mind mapping in the form of diagrams provided visual organisation, enabling identification of word associations and integration of themes. Transcription itself was used to dwell upon the data and contribute to interpretative activity [21], reinforced through repeatedly listening and reading the interview data. This enabled engagement with the phenomena and the experience of it by each individual, developing a deeper, richer understanding and interpretation of the data as themes emerged [17,23]. Layers of meaning and features of social interactions were recognised and used to further inform and enrich interpretation. The iterative process involved moving between transcripts, examining language and discourse, and gradually identifying themes, patterns, and shared meanings [21]. A second reviewer, TD, analysed 25% of transcripts and checked identified themes and clusters. Further secondary analysis was restricted by the richness of data and time limitations of the project. Any discrepancies were discussed and final themes agreed.

Field notes were analysed alongside interview data to contextualise the material and further develop the analytical process. Reflexivity was an important component of the process; the researcher’s perspectives and experiences enriched their interpretations in a co-construction of knowledge [19]. This was particularly important in light of the dual role held as both researcher and health professional. Recognising and exploring this role supported a transparent trail to demonstrate a clear connection between the data and interpretation, central to credibility in the context of a qualitative enquiry [16,24]. Engaging in the hermeneutic phenomenological method of enquiry enabled construction of rich, interpretative accounts of experiences to highlight prominent matters and concerns in practice.

## 3. Results

Four community healthcare workers were interviewed and they had a selection of roles:

- Clinical Psychologist
- Occupational Therapist
- Physiotherapist
- Therapy Support Worker

All participants had extensive knowledge and experience of working with individuals with MCI or dementia, with experience of working in their specialist field spanning from 3 years to over 20 years.

Four major themes were identified from the interview data, which are described below. Relevant quotes are presented to illustrate and support the themes, making direct links to the participants.

### 3.1. On-Going Assessment Is Important in Guiding Interventions and Influencing Change

Assessment was identified by participants as a key theme and basis to providing effective intervention and care. Participants identified standardised models of assessment as a means of evaluating function.

*‘I can take implications from standardised tests as to how the individual functions in real life… and that allows me to make links with the level of cognition in people and their mobility level’* [P2].

Assessment was also illustrated by participants as an on-going process in order to gain further insight into the context of a fluctuating and declining condition. This was described as being more difficult if the individual lived alone. Family and carers were found to be valuable sources of information and support, as well as presenting complex dynamics.

*‘Families, usually, in those circumstances are a really good source of collateral. However, sometimes…it can complicate the input’* [P4].

Participants perceived the assessment process as challenging and evolving. A range of assessment approaches, formats and models were reflected on as part of this process. Assessments were highlighted to include informal, perhaps more discrete assessment, to ensure interventions were person-centred.

Participants reflected on the value of assessments bringing about change. One participant discussed the ‘scope’ of influence and the opportunities to make improvements. Assessing the environment was a component of falls management which participants found important. Understanding the individual’s habits and routines was perceived as an essential component of this assessment.

*‘If you can make something an everyday activity by maybe re-arranging the environment so it becomes a more natural, more instinctive thing to do…. this hopefully takes them away from doing more risky activities’* [P3].

Participants discussed environmental adaptations made in response to visual and perceptual impairments.

*‘With perceptual difficulties in the home assessment…I am looking at the surfaces of the floor and the patterns…and if there’s anything that could be improved’* [P2].

Participants also felt that there were limitations to the changes they would like to make to benefit the individual. This included feelings of frustration at the limited time the participant might have with an individual. If you were ‘just visiting once a week, (you are) limited to how much influence and how much help you can be’ [P3]. Changes may not be sustained and individuals could be resistant to change as they may have had ‘feelings of autonomy about their own home and their own life’ [P3].

Participants highlighted the importance of individualised, thorough, on-going assessments to ensure interventions were at the right level for the right person and at the right time.

### 3.2. Knowledge and Experience Informs Practice

There were some interesting reflections on the incorporation of knowledge into practice. This included theoretical knowledge and the value of formal training, together with personal knowing based on personal experience and evidence-based care. Participants found that formal training gave them valuable knowledge for working with this population.

*‘Specific training on dementia and how mobility is affected in dementia because of the cognitive decline definitely helps’* [P2].

Participants identified their lack of knowledge and experience in relation to combining physical and cognitive strategies as part of falls prevention.

*‘I’m vaguely aware…that physical exercise is recommended for people to improve cognition. I know that cognition affects what the person can do physically. So training of that sort, I can see it as (being) successful or having impact’* [P2].

Limitations were also identified in incorporating such approaches and training, including time and resources. Participants related learning to team working as an additional means of developing and sharing skills. Joint working was described as “an excellent way of sharing knowledge and skills” [P3].

### 3.3. Individuals Living with Dementia Have Complex Physical and Cognitive Needs

Participants reflected on the physical and cognitive needs of individuals living with MCI or dementia and the associated complexity of risks. Physical decline was associated with the ageing process but also as a component of dementia. Cognitive domains, including executive function, were identified by participants as important contributors to falls.

*‘There are issues with perception and cognitive decline, not being able to anticipate the consequences of their actions…. it’s not just perception of environment, but also understanding. The awareness is not there’* [P2].

Participants highlighted a lack of insight and safety awareness as significant falls risk factors in this population. There could be a ‘lack of understanding that there is actually a hazard in front of them…that inability to recognise a possible trip hazard, and that inability to respond quickly enough to prevent the fall’ [P1]. Poor retention of information was also perceived as common in this population; this can lead to individuals being unable to recall or action safe techniques.

*…’because of the nature of dementia, people will do things which they think they can do but physically are not capable of…You may give advice, but whether they can remember that information is another question’* [P3].

Participants felt that the combination of physical and cognitive impairment has a significant impact on the individual and the risks involved. This was represented by one participant using visual imagery:

*‘If they’re not (stimulated) and not moving and so they are weak, they easily fatigue ….then, it’s a bit of a vicious spiral and….once you’ve stopped drinking and then you get a urine infection, then confusion, and everything sort of spirals out’* [P3].

Combining physical and cognitive training was perceived by participants as potentially beneficial but very complex, especially when exaggerated by components of emotion and anxiety as reflected by one participant:

*‘There can be more complexity to the cognitive aspect….if someone is highly anxious, there is a high probability that they will fall because it’s hard to manage your physical aspects if you feel like you’re flooding internally with fear and anxiety’* [P4].

Participants considered such dual-tasking as very demanding and could distract from focusing on safety. It could also result in poor compliance or resistance to interventions. Participants highlighted the need for a ‘graded approach…as we are all different’ [P1], and approaches need to be ‘very personalised’ [P2].

### 3.4. Teamwork Is Essential in Falls Prevention Strategies

Participants described the support offered through team working in this setting. Teams were defined in an extensive form, consisting of a variety of professionals, families, carers, organisations, and the individual service user.

Such a breadth of skill, knowledge and experience was felt by participants to be valuable within falls prevention work, due to the multitude of factors and the ‘wide scope’ [P3] of issues to consider.

Participants felt that multi-professional collaboration offered valuable sharing of information from a variety of perspectives to support individual service users.

*‘I have had really valuable discussions with other professionals and carers who have worked with service users I have worked with, and getting their perspective, because typically they are…getting different insights about the person’* [P4].

Participants reflected on how team working contributed to making key decisions and care planning within a holistic framework in collaboration with the service user and their family.

There were also some interesting reflections on the complex dynamics and conflicts that can occur within multi-disciplinary teams. This included different goals or objectives, such as carers or family members who may not facilitate functional independence of the individual in the way a therapist would. There were also some frustrations around perceptions of different professional roles, which delayed referrals. The role of physiotherapy in dementia and how it relates to falls, for example, can be ‘misunderstood or underestimated’ [P1], which can delay valuable and timely input.

## 4. Discussion

### 4.1. Summary of Findings

This study provides valuable insights into the challenges experienced by community health care workers when managing falls for individuals living with MCI or early dementia. To the best of our knowledge this is the first qualitative study to explore practice perceptions on this subject. This study found that combining physical and cognitive strategies was not widely practised as part of a falls prevention strategy. Such an approach has potential benefits but was highlighted as being complex and needs to be person-centred.

Participants identified impaired executive function as an important factor when managing falls in this population. This reflects findings from other studies [11,25,26]. There is a growth in evidence that associates executive function with gait changes and balance as important factors contributing towards falls risk. Adults with cognitive impairment have been found to demonstrate altered gait patterns, including reduced speed and increased stride variability, together with other motor dysfunctions such as reduced balance. The role cognition plays in the control of gait and balance can be considered to be dementia-specific falls risk factors, placing this population at a much higher risk of falls [26]. Additionally, a systematic review investigated specific cognitive factors to gait, balance, and falls, which further demonstrates the association between impaired executive function and gait changes [11]. This link contradicts earlier views that falls are a result of motor function failures [25]. Further studies found that regions of the brain involved in cognitive functioning and memory are required to coordinate mobility, balance, and gait [26]. Training both physical and cognitive functions has the potential to prevent mobility limitations and falls in dementia [26,27].

Dementia involves the progressive loss of an array of executive functions. In addition to physical alterations, it also affects movement coordination, visuospatial perception, and ability to recognise or respond to hazards [7]. In our study, participants experienced such challenges in practice, highlighting the higher level executive skills including flexible thinking and ability to adapt, which must be considered. Our study supports findings where an association was found between impaired gait adaptability and impaired executive function [28]. A further study found that falls with declining executive function are particularly relevant to frail older adults as they may not have the cognitive abilities to compensate for physical deficits [11]. This raises the question as to whether cognitive risk factors for falls can signify an emerging dementia pre-diagnosis, for which further research is required. Kearney et al. [11] adopted a rigorous design but reliability of their findings was limited by a lack of uniformity in outcome measures. There was also limited consensus regarding a definition of executive function, but this is found throughout the literature so comparisons between studies are challenging. There are also few studies that specifically include or focus on adults with cognitive impairment [29], reflecting complex and highly regulated approval processes, which lead to the under-represented populations in health research [30].

The different levels of physical and cognitive abilities are important factors when considering combined approaches. Individuals who are further advanced in their dementia would find such combinations more challenging and potentially over-whelming, to the extent that it could be detrimental. This is reflected in the findings of our study, particularly in the imagery of ‘flooding’ used by one participant which conveyed a feeling of submersion, empathising with the strength of emotion which can impact such individuals when faced with physical and cognitive challenges simultaneously. High levels of anxiety and emotion can increase falls risks, and makes addressing physical deficits more challenging. This highlights the value and importance of experience and incorporating knowledge into practice, as illustrated in the findings of our study.

Dual-tasking can be extremely challenging and emphasises the need for on-going assessments to be person-centred. In our study, participants identified on-going assessment as a basis for clinical decision-making and determining what approaches were appropriate or beneficial to the individual. Different models of assessment were illustrated by participants as being crucial to gaining insight and adding context to dementia as a complex and deteriorating condition. This key theme is in line with previous studies. For example, Caetano et al. [28] found that the addition of a cognitive task to a physical task resulted in the individual prioritising the physical task by slowing the gait speed, for example. Individuals who are further impaired cognitively may not be able to perform such prioritisation and are at a higher risk of falls. This reinforces the need for careful consideration of the stage of dementia the individual has reached. It also supports the important role of earlier intervention and how this affects outcome [31]. Early interventions in falls prevention specifically designed for this population supports longer-term sustainability of any changes, helping to reduce dependency and prevent crisis [2,8]. The complex interplay between motor and cognitive functioning is common in daily activities [32]. When introduced in the earlier stages of dementia, combining physical and cognitive approaches can improve dual-task capabilities. Such an approach helps to inform and achieve meaningful, functional goals [9] to be important to the individual, to their carers and family and to the health care worker, as identified in our study. The findings from our study supports those from a previous study where significant improvements were found in cognitive functions, functional balance, and functional status in individuals with MCI who underwent a 10-week structured functional task exercise programme [14]. Additionally, a stimulating environment can enhance the benefits of a combined approach and increase the impact on neuroplasticity [33,34]. This was highlighted in our study by participants who felt that a lack of stimulation affects motivation and leads to decreased activity.

Consideration of the stage of dementia can also inform intervention choice such as exercise mode or approach. Multicomponent exercise training, for example, can improve functionality in older people by addressing several falls risk factors associated with physical decline in older age [10]. This includes improvements in strength, flexibility, and balance which in turn improves physical function and reduces the risk of falls [10,35]. However, which exercise mode most suitable for older adults, particularly those with dementia, requires further research [36,37]. Previous studies found that multicomponent exercise can benefit individuals with dementia in improving function [37]. Our study also highlights the challenges dual-tasking can present for individuals in the more advanced stages of dementia; combining different exercise modes simultaneously could be over-whelming and potentially distressing for such individuals and further research is required [37]. Exploring exercise training modes in the community is limited by the need for more specialised exercise health professionals and is beyond the scope of this study.

Participants emphasised the strength and support provided as part of a multi-disciplinary team, particularly reflecting on the breadth of skill and experience required in the context of dementia care. The sharing of knowledge from experience is illustrated as being essential to applying, effectively, formal learnings and theory to practice. Previous studies have also found that an integrated team approach enhanced intervention and progression towards goals [38]. However, issues of conflict and potential for dilution of professional identity and role were also raised. This reflects the wider changes to healthcare with the growth of the Integrated Care System (ICS) which is recognised as a response to the rising numbers of older people living with complex long-term health conditions [39]. This move is a key part of the NHS Long-Term Plan to deliver person-centred care that is effective, timely, and equitable [40]. Integrated care is complex and there is a lack of evidence or guidance on how professionals can prepare themselves to preserve their professional identity while working as an effective team member [41]. Identities must be flexible and able to adapt to a changing professional landscape within an on-going process of identity formation. Findings in our study highlight the lack of empirical research relating to workforce perceptions of an integrated care system [39]. Identifying what matters to the workforce would enable systems to respond, adapt and deliver optimum sustainability to very vulnerable populations.

### 4.2. Strengths and Limitations

Reflexivity was employed throughout the research process as a strategy for ensuring rigour and trustworthiness. The study design enabled transparency of the researcher role to promote accuracy and credibility of findings. A diversity of perspectives was included, and the four major themes were identified in all the interviews; this further enhances credibility. A limitation of the study is the small sample from one community therapy team which may affect generalisability due to chance differences between regions, cultures, and wider populations. Nevertheless, the focus of a qualitative exploration is the rich, volume intensive data rather than a large sample. Researcher professional links to the community therapy team may have impacted the researcher’s interpretation and selection of information provided to them. Interpretation is subjective but the pre-existing knowledge and insight around the setting and phenomena under investigation, embodied in the researcher’s position as an insider, means the researcher was sensitised to specific literary or dialectic dimensions and underlying meanings of the data. A second researcher also analysed one of the interviews to increase the rigour.

## 5. Conclusions

The findings of this study contribute to the under-developed evidence-base for the qualitative exploration of key health worker’s lived experience in caring for individuals with dementia in the community. Four major themes were developed from interview data which highlighted the potential benefits of combining physical and cognitive training, but also that falls prevention in this context is multifactorial and complex.

Based on participant responses in this study, the integration of cognitive and physical training into practice in this setting offers the potential benefits of reducing the risk of falls and optimising function. Findings highlight the importance of considering the stage of dementia. Early intervention is more likely to bring about sustainable changes such as maintaining functional independence for longer. It is also suggested that clinicians take account of the level of physical ability, frailty, and function the individual has reached to determine whether dual-task training is appropriate and beneficial.

Further research is required to explore higher cognitive functions, for example, and how this relates to gait, balance and falls. This study highlighted the need to investigate further the relationship between adaptability and executive function to inform practice. Specific, evidence-based guidelines are required, which include the risk factors unique to this population. Further qualitative exploration of key stakeholder perspectives would contribute valuable empirical evidence to inform such policy development.

The findings also raised the importance of the emotional aspects, which can impact the effect of interventions when working with this population. Current evidence is lacking in this area. Emotion and anxiety can make dual-tasking more challenging, even detrimental, and so a person-centred, team-based approach is essential, supported by regular assessments and patient knowledge.

## Figures and Tables

**Table 1:** Phases of IPA Analysis.

Phase	Task
1	Build familiarity with the data through immersion, closely examining and evaluating the data, exploring the semantic content.
2	Identification of issues, patterns and emerging themes.
3	Searching for connections between issues and connecting them into clusters, based on their similarities. Looking for patterns across cases, deepening the analysis and generation of interpretations.
4	Using iterative analysis, the themes are developed and related to the primary material (the transcripts).
5	Presentation and dissemination of the interpretation.

## Data Availability

Data are contained within the article or Supplementary Materials.

## Supplementary Materials

The following supporting information can be downloaded at: https://www.mdpi.com/article/10.3390/geriatrics7050113/s1, Table S1: Interview Schedule; Table S2: Thematic Analysis Flow Chart.
