# Supplementary material for: The Lived Experience of Healthcare Workers in Preventing Falls in Community Dwelling Individuals with Dementia
Source: Geriatrics (Basel). 2022 Oct 7;7(5):113. doi: 10.3390/geriatrics7050113 (PMC9601408; doi:10.3390/geriatrics7050113)
Supplement: Supplementary file 1 [file geriatrics-07-00113-s001.zip › Supplementary Files.pdf]

Supplementary Files

Table S1

Interview Schedule

	Question and prompts	Comments/ observations
1.	How much of your caseload includes individuals affected by cognitive impairment and/or early dementia?	
2.	Are there any particular challenges or obstacles you encounter when working with this population?<br>• Lack of resource<br>• Time constraints<br>• Lack of understanding or retention of information<br>• Lack of support (family members, organisation)<br>• Lack of training or knowledge	
3.	Are there any facilitators when working with this population?<br>• Training/learning<br>• Experience<br>• Joint working<br>• Supportive family/friends network	
4.	Do you find that falling is an issue for this population?<br>• Different types of falls<br>• Consequences of falls	
5.	What do you find are the main reasons for falling in this population?<br>• Cognition<br>• Anxiety/fear<br>• Physical limitations<br>• Lack of insight<br>• Environment<br>• Self-neglect	
6.	What kind of interventions have you used or tried to prevent falls in this population?<br>• Cognitive strategies<br>• Physical strategies<br>• Environmental<br>• Family support<br>• Combining physical and cognitive strategies	
7.	What do you think might be the more effective intervention/s to prevent falls in this population?<br>• Combining physical and cognitive strategies<br>• What aspect to focus on<br>• Joint working	

Are there any other issues or comments you would like to make which you feel is important which we have not talked about?

Table S2
Thematic analysis – Flow Chart

Issues	Clusters	Themes
Assessment<br>Change<br>Environment<br>Knowledge<br>Time<br>Physical<br>Cognition<br>Collaboration<br>Support<br>Risk*<br>Person-centred*	Assessment<br>Change<br>Environment<br>(Risk)*<br>(Person-Centre)*	On-going assessment is important in guiding interventions and influencing change
	Knowledge<br>Time<br>(Risk)*<br>(Person-centred)*	Knowledge and experience informs practice
	Physical<br>Cognition<br>(Risk)*<br>(Person-centred)*	Individuals living with dementia have complex physical and cognitive impairments
	Collaboration<br>Support<br>(Risk)*<br>(Person-centred)*	Team-work is essential within falls prevention work

*Wider issues which fed into more than one cluster
